# Altered plasma fatty acids composition in autism spectrum disorder: a case-control study

**DOI:** 10.3389/fpsyt.2025.1627704

**Published:** 2025-09-24

**Authors:** Ye Liu, Lili Zhang, Su Wang, Shasha Guo, Zhongbi Peng, Zhaojing Tai, Yun Chen, Hao Zhou

**Affiliations:** ^1^ Department of Otolaryngology, Guizhou Provincial People’s Hospital, Medical College of Guizhou University, Guiyang, China; ^2^ Department of Pediatrics, Minhang Hospital, Fudan University, Shanghai, China; ^3^ Department of Pediatrics, Guizhou Provincial People’s Hospital, Guiyang, China; ^4^ Department of Neurorehabilitation, Pediatric Cardiovascular Center of Guizhou Hospital for Shanghai Children Medical Center, Guiyang, China; ^5^ Department of Rehabilitation, Children’s Hospital of Fudan University, National Children’s Medical Center, Shanghai, China

**Keywords:** autism spectrum disorder, fatty acids, saturated fatty acids, unsaturated fatty acids, receiver operating characteristic curve

## Abstract

**Background:**

Due to the variability in clinical manifestations and the frequent diagnostic delays associated with autism spectrum disorder (ASD), interest in identifying fatty acids as potential biomarkers is increasing. Nonetheless, owing to inconclusive evidence, further investigation is needed.

**Objective:**

To explore the relationship between fatty acids and ASD risk and identify distinct fatty acid metabolites in children with ASD.

**Methods:**

Plasma fatty acid levels were tested in totally 131 participants (ages 2–6 and male-to-female ratio 2.5:1) with and without ASD using gas chromatography coupled to flame ionization detector and mass spectrometer (GC-FID/MS) technology. Between-group differences in each fatty acid and the omega-3 polyunsaturated fatty acid/arachidonic acid (AA) ratio were explored. We adjusted for covariates via multivariable models. The discriminatory sensitivity of meaningful fatty acids between ASD and control groups was assessed via receiver operating characteristic curve (ROC) analysis.

**Results:**

Two of 22 fatty acids significantly differed between children with ASD and typically developing children. Specifically, C20:4ω6 (AA) (457.4 ± 195.3 μmol/L *vs.* 493.3 ± 111.9 umol/L, *P* = 0.044) and C24:0 (34.7 ± 7.9 μmol/L *vs*. 38.3 ± 8.7 μmol/L, *P* = 0.019) levels were significantly lower in the autism group than in the control group, whereas the alpha-linolenic acid (ALA)/AA ratio [0.13(0.10, 0.18) *vs.* 0.10(0.08, 0.15)] was significantly greater in children with autism than in those without. Potential interactive effects between AA, C24:0, ALA/AA and gastrointestinal syndromes were further observed. Biomarkers were assessed via ROC analysis, which revealed AA, C24:0 and ALA/AA AUC values of 0.60(0.50~0.70), 0.62(0.52~0.72) and 0.62 (0.52~0.71), respectively.

**Conclusions:**

Fatty acid disturbance was observed among children with ASD, particularly in terms of AA, C24:0 and the ALA/AA ratio. These findings provide valuable insights into the underlying mechanisms of ASD and suggest that modulating fatty acid levels could serve as an intervention strategy.

## Introduction

Autism spectrum disorder (ASD) is a complex neurodevelopmental condition characterized by challenges in social interaction, communication, and repetitive behaviors (1). The prevalence of autism is sharply increasing with a current global prevalence rate of 1% (2); however, it affects 0.7% of children in China (3), significantly affecting families and society. Despite extensive research, the etiology of ASD remains largely elusive, with genetic, environmental, and biological factors all being implicated in its development (4). Recent studies have increasingly focused on the role of metabolic and nutritional imbalances in ASD, with metabolic factors being viewed as the bridge between genetic susceptibility and environmental factors (5).

Emerging evidence indicates that children with ASD may exhibit altered fatty acid profiles, including imbalances in omega-3 and omega-6 polyunsaturated fatty acids (PUFAs) (6–8). These imbalances could contribute to the neurological and behavioral symptoms observed in individuals with ASD. Specifically, deficiencies in omega-3 fatty acids, such as eicosatetraenoic acid (EPA), docosahexaenoic acid (DHA) and alpha-linolenic acid (ALA), have been associated with increased inflammation and impaired neurodevelopmental outcomes (9). Conversely, excessive levels of omega-6 fatty acids may exacerbate inflammatory pathways, further complicating the clinical presentation of ASD (10).Furthermore, some dietary interventions aimed at modifying fatty acid intake have shown promise in improving behavioral and cognitive outcomes in individuals with ASD (11, 12).

Despite these findings, we identified two major gaps in the literature. First, there are inconsistencies across studies regarding the specific abnormalities in fatty acid levels observed in individuals with ASD. Some studies have reported significantly lower concentrations of C20:5ω3 and C18:2ω6 than in the control group (6), whereas others have reported no significant difference between groups (13). Second, previous studies have focused on a relatively narrow range of fatty acids, primarily unsaturated fatty acids, which cannot fully reflect the overall status of fatty acid metabolism (14).

In this study, we aimed to investigate the broader fatty acid profile in autistic children to understand the related fatty acid metabolism levels. By employing gas chromatography coupled to flame ionization detector and mass spectrometer technology, we sought to characterize the fatty acid composition and the omega-3 PUFAs/arachidonic acid (AA, C20:4ω6) ratio in detail. Through this research, we hope to contribute to the growing body of knowledge on the metabolic underpinnings of ASD and highlight the potential of fatty acids as modulators of neurodevelopmental health. Based on compelling prior evidence implicating specific fatty acid imbalances in ASD (15–17), this study was hypothesis-driven, primarily focusing on AA, C24:0, and the ratio of alpha-linolenic acid to AA (ALA/AA) as pre-specified targets of interest. The examination of the broader fatty acid panel is presented for exploratory purposes to provide a comprehensive metabolic context.

## Methods

### Participants

From December 2023 to December 2024 a total of 70 children diagnosed with ASD from Guizhou Provincial People’s Hospital and 61 typically developing (TD) were enrolled in this study. Eligible children with ASD met the following criteria: 1) had a diagnosis of ASD confirmed by a qualified and experienced psychiatrist on the base of the criteria of the Diagnosis and Statistical Manual of Mental Disorders, Fifth Edition; 2) aged between 2 and 6 y at recruitment; and 3) had parents or legal guardians who were willing to allow their children to participate and provide plasma samples. The exclusion criteria included 1) symptomatic autism (autism-related disorders, such as Rett syndrome and fragile X syndrome); 2) mental illness (primary mental illnesses, such as schizophrenia and bipolar disorder); 3) neurological disease; 4) metabolic disorders; 5) essential fatty acid supplementation before or at the time of sample collection. In addition, a total of 61 TD children undergoing preschool entrance physical examination were recruited from the physical examination clinic at Guizhou Provincial People’s Hospital. The inclusion criteria for the TD group were age-matched to the ASD group but without any mental, neurological, or metabolic disease. As shown in **Table 1**, the two groups had comparable age and BMI distributions (*P* >0.05), supporting selection validity.

**Table 1:** Basic characteristics of the participants.

Characteristics	Control(n=61)	ASD(n=70)	*χ* ^2^/*t* value	*P* value^1^
Sex(male:female)	33:28	61:9	17.562	<0.001
Age(y)^2^	3.7 ± 1.0	3.5 ± 1.4	0.857	0.393
BMI^2^	15.3 ± 0.2	15.5 ± 0.2	-0.686	0.491
Parental education			0.645	0.721
Postgraduate	9(14.8%)	14(20.0%)		
Bachelor	26(42.6%)	27(38.6%)		
Undergraduate	26(42.6%)	29(53.4%)		
Maternal education			1.006	0.605
Postgraduate	7(11.5%)	5(7.1%)		
Bachelor	25(41.0%)	27(38.6%)		
Undergraduate	29(47.5%)	38(54.3%)		
Parental occupation			1.980	0.372
Civil servant	9(14.8%)	5(7.1%)		
Tech worker	27(44.3%)	34(48.6%)		
Freelancer	25(40.9%)	31(44.3%)		
Maternal occupation			1.547	0.462
Civil servant	8(13.1%)	6(8.6%)		
Tech worker	20(32.8%)	19(27.1%)		
Freelancer	33(54.1%)	45(64.3%)		
Residence			2.387	0.122
Rural	15(24.6%)	26(37.1%)		
Urban	46(75.4%)	44(62.9%)		
Any GI symptom(yes,%)	19(31.2%)	35(50.0%)	4.781	0.029
Medicine use	0	11.4%	–	–
Special diet	0	0	–	–
Nutritional supplements	0	41(58.6%)	–	–
ASRS score^2^	–	122.3 ± 19.2	–	–

ASD, autism spectrum disorder; ASRS, Autism Spectrum Rating Scale.

^1^Performed by 2-sample t-test for normally distributed variables and chi-square test for categorical variables.

^2^values are the mean ± SD.

### Ethical approval

The study was conducted according to the guidelines of the Declaration of Helsinki and approved by the Ethics Committee of Guizhou Provincial People’s Hospital (No. 2023-046,2023.6.2).

### Clinical data collection

Upon enrolment, demographic information, which included variables such as age, sex, height, weight, parental education level, and parental occupation, was collected via self-administered questionnaire. Additionally, data regarding medication usage, adherence to specialized diets (including therapeutic diets such as ketogenic, gluten-free, or low-protein diets, as well as dietary patterns influenced by familial or religious beliefs, such as vegetarian or pork-free diets), GI symptoms, and nutritional supplement intake were gathered through clinical interviews.

The symptoms of autism were evaluated using the Autism Spectrum Rating Scale (ASRS) total score for participants diagnosed with ASD. The ASRS is a tool with established reliability and validity in China (18). GI symptoms were defined as the presence of ≥1 of the following within the past 3 months: abdominal bloating, constipation, vomiting, abdominal pain, or diarrhea.

### Biosample collection

Three milliliters blood samples were collected in the morning from children who fated overnight (12 hours); EDTA was used as an anticoagulant. The plasma was immediately separated via centrifugation at 3000×g for 10 minutes at 4 °C. The obtained plasma samples were aliquoted into 5 Eppendorf tubes (1.5 mL), with 200 μL per tube, and frozen at -80 °C until analysis. The metabolomics analysis was performed within a storage period of 3 mo.

### GC–FID/MS methodology

Fatty acid profiling was detected using an Agilent 7890B gas chromatography coupled to an Agilent 5977B mass spectrometer with a flame ionization detector (Agilent Technologies, USA). Data collection and analysis were conducted using Mass Hunter software (Version B.08.00, Agilent, USA), with retention times compared to qualitative standards, and verification using mass spectra. Quantification was performed using the internal standard method. Peak determination and peak area integration were performed with MassHunter Workstation software (Agilent, Version B.08.00). Methylated fatty acids were identified by comparing with a chromatogram from an initial mixture of 37 known standards and further confirmed with their mass spectral data. The concentration of individual fatty acid was calculated from the FID data related to the internal standard, as previously reported (19). Detailed descriptions of reagent specifications, metabolite extraction procedures, GC-FID/MS analytical conditions and the list of the fatty acid standards are provided in **Supplementary File 1**.

A total of 37 fatty acids were initially targeted for analysis using GC-FID/MS, of which 15 were not included in the final analysis due to technical limitations and biological characteristics. The specific reasons are as follows: (1) biological limitations of odd-chain fatty acids: Mammalian fatty acid metabolism follows the acetyl-CoA metabolic pathway and cannot naturally synthesize odd-chain fatty acids. Only C15:0 and C17:0 can be detected through dietary intake, hence no other odd-chain fatty acids were found in the samples; (2) detection sensitivity threshold: some fatty acids concentrations were below the instrument’s detection limit or signal-to-noise ratio, making accurate quantification impossible; (3) volatile loss of fatty acids: Short-chain fatty acids with carbon chain lengths of <12 could not be retained due to their low boiling point characteristics during the formaldehyde fixation process.

### Statistical analysis

Descriptive statistics were performed in total and by group. The characteristics of the study participants, including all fatty acids, are presented as the means (SDs) for normally distributed variables, medians (IQRs) for skewed variables and frequencies with percentages for categorical variables. Group comparisons were performed by 2-sample t tests, Two-sample Wilcoxon rank-sum (Mann–Whitney) tests and chi-square tests. Linear regression models were performed, with fatty acids showing significant group differences (P < 0.05) as the outcome variable and group (TD group compared with the ASD group) as the exposure variable. To reduce confounding bias, multivariable linear regression models were performed to estimate coefficients with 95% confidence intervals (CIs) adjusted for age (y), sex, BMI z-score, parental education level (postgraduate, bachelor, undergraduate), parental occupation (civil servant, tech worker, freelancer), and GI symptoms (yes, no). Because poor dietary behaviors are common in children with ASD, they may cause an imbalanced intake of fatty acid nutrients and bias the association. To further support these findings, multivariable linear regression models, with or without interaction terms (group×GI symptoms) were performed after adjusting for the abovementioned confounders. Estimated margins with 95% CIs and Wald tests comparing groups stratified by GI symptoms (yes or no) are reported. To evaluate the biomarkers for ASD, receiver operating characteristic (ROC) analysis was performed.

The primary, hypothesis-driven analyses focused on AA, C24:0, and the ALA/AA ratio, based on strong prior evidence (15–17). The analyses of the remaining fatty acids are considered exploratory. Consequently, multiple-testing correction was not applied to the primary targets, while the exploratory findings should be interpreted with caution.

## Results

### Characteristics of participants

In total, 70 children with ASD and 61 TD children participated in this study. The average age in the ASD group was 3.5 ± 1.4 y, whereas it was 3.7 ± 1.0 y in the TD group. Sociodemographic characteristics were generally similar between the 2 groups, except that unlike TD children (54.1%), a very large proportion of children diagnosed with ASD were boys (87.1%). A higher prevalence of GI symptoms was observed in ASD patients, with an ASRS score of 122.3 ± 19.2. The detailed information is shown in **Table 1**.

### Differences in fatty acid profiles between groups

Most of the saturated fatty acids (SFAs) and unsaturated fatty acids (UFAs) were present at lower levels in the ASD group than in the TD group. Notably, the levels of AA (457.4 ± 105.3 µmol/L *vs*. 493.3 ± 111.9 µmol/L, *Z=*2.012*, P=*0.044) and C24:0 (34.7 ± 7.9 µmol/L *vs*. 38.3 ± 8.7 µmol/L, *Z=*2.339*, P=*0.019) were significantly lower in the ASD group than in the control group. The ratio of ALA/AA [0.13(0.10, 0.18) *vs.* 0.10(0.08, 0.15), *Z* = -2.293, *P* = 0.022] was significantly greater in the ASD group than in the control group (**Table 2**). The results from the multivariable regression models suggested similar trends (**Table 3**). However, the linear regression analysis indicated no significant association between the described fatty acids and the ASRS scores (P>0.05).

**Table 2:** Fatty acid concentrations and ratios between the ASD and TD groups.

Variables	TD(n=61)	ASD(n=70)	*Z* value	*P* value^1^
Unsaturated fatty acids (UFAs)	5318.2(4721.4, 5954.3)	5315.9(4661.1, 6044.8)	-0.014	0.989
Polyunsaturated fatty acids	3481.5(3199.7, 3734.8)	3383.8(3090.6, 3789.3)	0.448	0.654
Omega-3 series				
C18:3ω3(ALA)	51.1(42.1, 65.6)	56.4(42.5, 79.6)	-1.297	0.195
C20:5ω3(EPA)	16.0(10.0, 22.9)	15.9(10.6, 24.5)	0.000	1.000
C22:6ω3(DHA)	99.8(72.2, 124.7)	96.4(68.8, 123.2)	0.291	0.771
Omega-6 series				
C18:2ω6	2670.2(2413.6, 2895.3)	2630.5(2364.4, 2959.3)	0.069	0.945
C18:3ω6	22.8(18.4, 29.8)	24.8(17.7, 38.9)	-0.771	0.441
C20:2ω6	17.2(14.9, 19.5)	16.8(15.0, 19.4)	-0.171	0.864
C20:3ω6^2^	81.4 ± 19.8	78.9 ± 21.6	1.315	0.189
C20:4ω6(AA)^2^	493.3 ± 111.9	457.4 ± 105.3	2.012	**0.044**
Ratio of ω-3 PUFAs/AA				
ALA/AA	0.10(0.08, 0.15)	0.13(0.10, 0.18)	-2.293	**0.022**
EPA/AA	0.03(0.03, 0.05)	0.04(0.02, 0.05)	-0.669	0.504
DHA/AA	0.19(0.17, 0.24)	0.21(0.16, 0.26)	-0.849	0.396
Monounsaturated fatty acids (MUFAs)	1856.1(1516.1, 2160.3)	1894.2(1544.4, 2211.4)	-0.738	0.460
C16:1	117.7(94.8, 149.0)	117.16(91.0, 147.8)	0.503	0.615
C17:1	10.7(9.2, 12.7)	11.2(8.3, 13.2)	-0.180	0.857
C18:1ω9	1615.8(1336.3, 1885.5)	1650.0(1353.6, 1978.8)	-0.798	0.425
C20:1	13.5(10.3, 16.2)	14.6(10.7, 17.4)	-1.126	0.260
C24:1^2^	83.1 ± 22.3	81.5 ± 18.3	-0.009	0.993
Saturated fatty acids (SFAs)	2917.3(2497.5, 3364.3)	2894.5(2499.4, 3289.7)	0.189	0.850
C12:0	14.0(10.0, 27.3)	15.4(9.7, 23.2)	0.046	0.963
C14:0	63.8(49.0, 103.9)	76.1(50.8, 97.0)	-0.526	0.598
C15:0	14.1(11.6, 17.4)	13.9(9.7, 18.2)	0.563	0.573
C16:0	2075.4(1759.4, 2372.3)	2052.4(1777.7, 2347.5)	0.014	0.989
C17:0	17.4(14.9, 20.3)	16.9(14.2, 20.5)	0.411	0.681
C18:0	630.0(543.0, 686.1)	597.9(526.9, 665.8)	0.937	0.348
C20:0	19.0(17.6, 21.1)	18.2(16.2, 20.0)	1.601	0.109
C22:0^2^	46.0 ± 8.8	43.0 ± 9.2	1.943	0.052
C24:0^2^	38.3 ± 8.7	34.7 ± 7.9	2.339	**0.019**

TD, typically developing; ASD, autism spectrum disorder; ASRS, Autism Spectrum Rating Scale.

^1^Two-sample Wilcoxon rank-sum (Mann-Whitney) test.

^2^The values are means (± SDs).

Bold values indicate statistically significant differences (P < 0.05).

**Table 3:** Adjusted estimates of metabolites and interactive effects with GI symptoms (yes, no).

	Without interaction		With interaction					
	Mean difference (95% CI)		Mean difference (95% CI)		Predictive margins (95%CI)			
	Group(1) *vs.* Group(0)		Group(1) *vs*. Group(0)		Group 0 (n=61)		Group 1 (n=70)	
	Adjusted^1^	P>|t|	Adjusted^2^	P>|t|	GI(no)	GI(yes)	GI(no)	GI(yes)
AA	-49.12(-90.79, -7.46)	0.021	-52.53(-105.55, 0.49)	0.052	492 (455, 529)	493(443, 544)	439(395, 483)	449(408, 490)
C24:0	-3.94(-7.04, -0.84)	0.013	-3.19(-7.02, 0.83)	0.111	38.4(35.7, 41.2)	38.8(35.1, 42.6)	35.2(32.0, 38.5)	33.8(30.7, 36.8)
ALA/AA	0.08(0.01,0.14)	0.017	0.07(-0.01, 0.15)	0.099	0.12(0.07, 0.17)	0.13(0.05, 0.20)	0.19(0.12, 0.25)	0.22(0.16, 0.28)

AA, typically developing; ASD, autism spectrum disorder; ASRS, Autism Spectrum Rating Scale.

^1^Estimated from multivariable regression without an interaction term, adjusted for age (years), sex, BMI z-score, gastrointestinal (GI) symptoms (yes, no), and the first two principal components derived from multiple correspondence analysis of parental educational attainment (Postgraduate or above, Bachelor, College or below) and parental occupational categories (Civil Servant, Tech Worker, Freelancer).

^2^The results were estimated via multivariable regression with an interaction term between group and GI symptoms, adjusted for age (years), sex, BMI z-score, gastrointestinal (GI) symptoms (yes, no), and the first two principal components derived from multiple correspondence analyses of parental educational attainment (Postgraduate or above, Bachelor, College or below) and parental occupational categories (Civil Servant, Tech Worker, Freelancer).

### Interactive effects on GI symptoms

**Figure 1:**
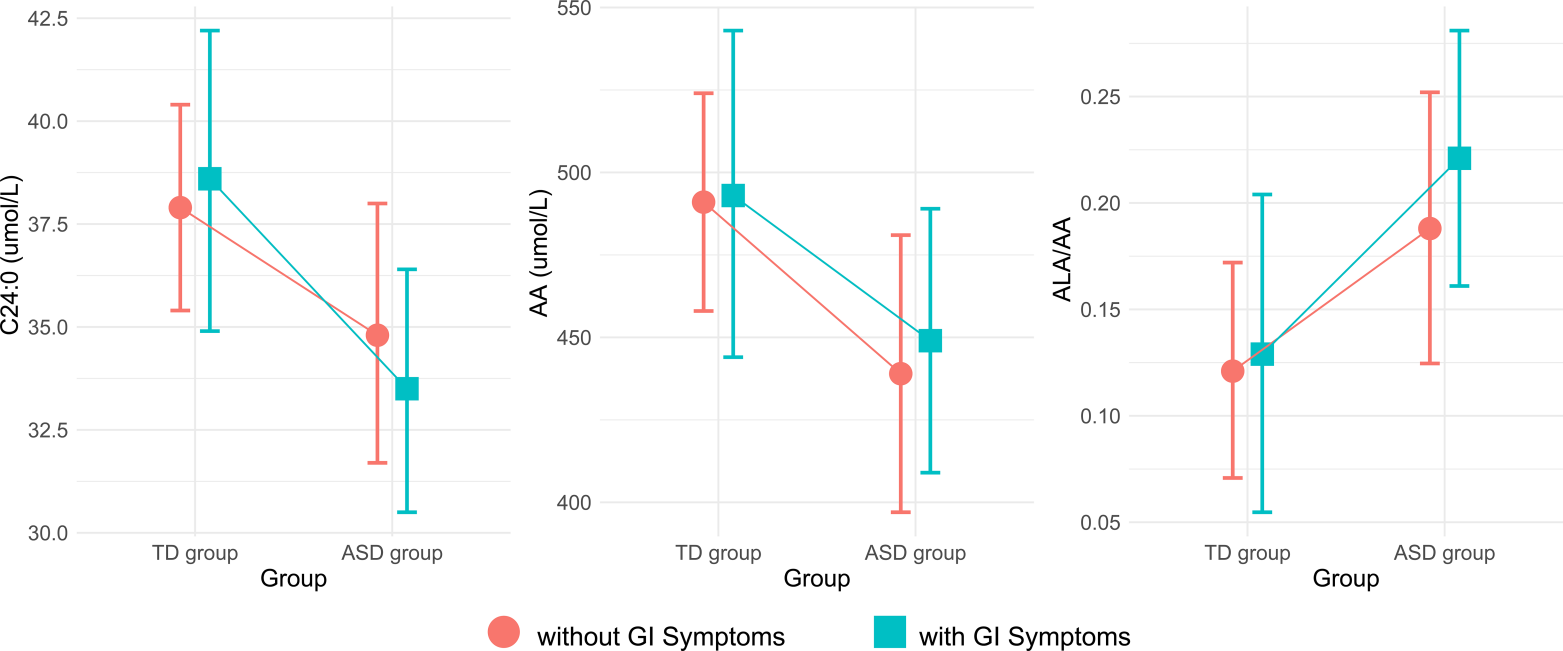
Plots of estimated margins between the TD and ASD groups and their interaction with GI symptoms. TD, typically developing; ASD, autism spectrum disorder; GI, gastrointestinal; AA, arachidonic acid; ALA, alpha-linolenic acid.

Analysis of predictive margins across different groups and gastrointestinal (GI) symptoms suggested potential interactions involving GI symptoms for AA, C24:0 and the ALA/AA ratio (**Figure 1**). Upon incorporating the interaction term (Group×GI symptoms) into the model, a significant difference was not observed in AA, C24:0, or ALA/AA levels (**Table 3**). However, Wald’s test still revealed the significant effect of groups on C24:0 (F = 6.573, *P=*0.012), AA (F = 5.104, *P=*0.026) and the ALA/AA ratio (F = 6.028, *P* = 0.016).

### Potential biomarkers

**Figure 2:**
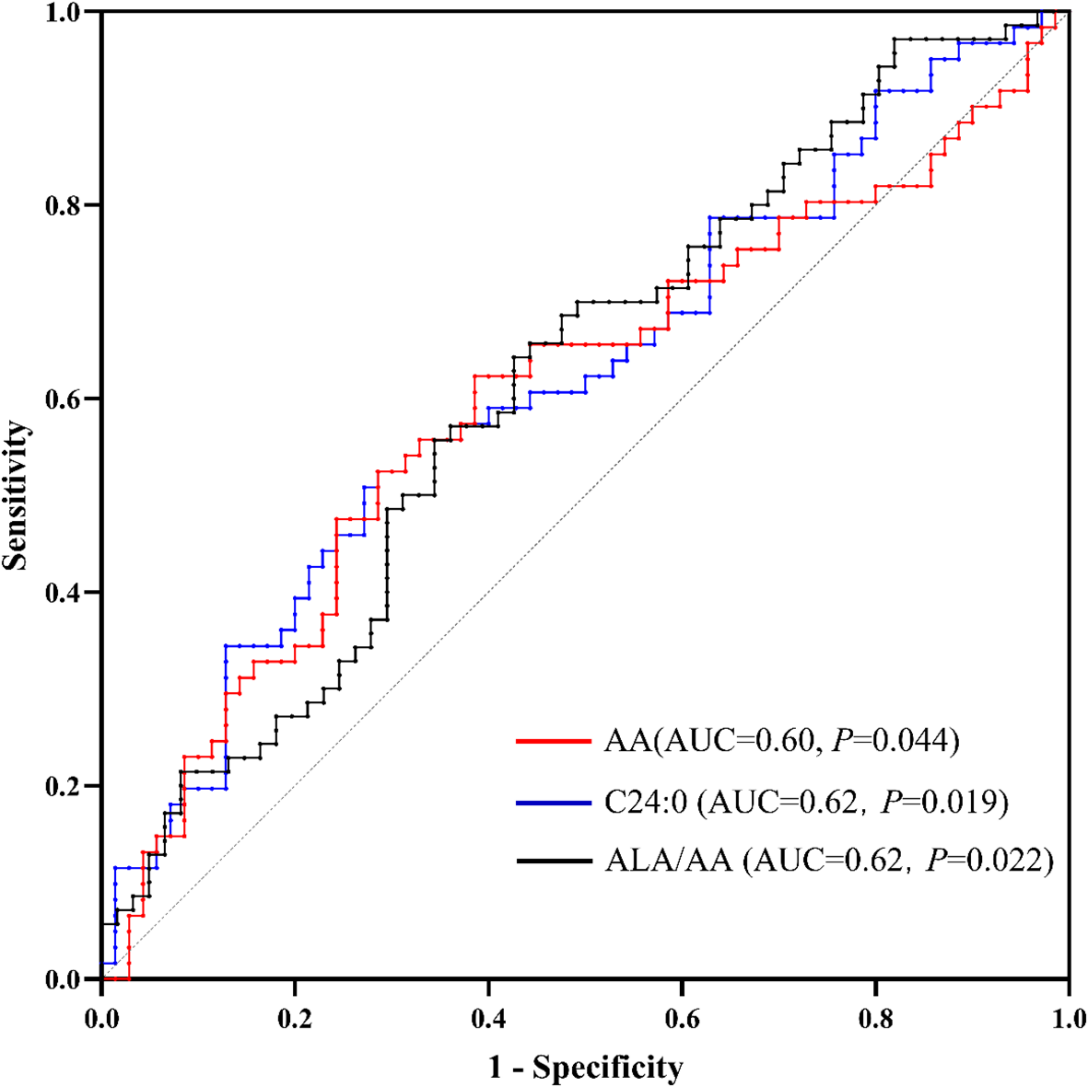
Receiver operating characteristic (ROC) curves for AA, C24:0 and the ALA/AA ratio as biomarkers for autism. AA, arachidonic acid; ALA, alpha-linolenic acid.

We employed ROC analysis to quantify the discriminative capacity of these biomarkers between ASD and control groups, calculating area under the curve (AUC) values with 95% confidence intervals (*P* < 0.05, **Figure 2**). The AUC values for AA, C24:0 and ALA/AA were 0.60(0.50~0.70), 0.62(0.52~0.72) and 0.62 (0.52~0.71), respectively.

## Discussion

In this study, we observed a significant reduction in both AA, an unsaturated fatty acid, and C24:0, a saturated fatty acid, in children with autism compared with the control group. Furthermore, we found a significantly increased ratio of ALA/AA, which is one of the omega-3 PUFA/AA ratios. These findings align with a growing body of literature that identifying disturbances in fatty acids metabolism as a feature of ASD (17, 20, 21). Notably, we observed that these fatty acid levels were influenced by the presence of GI symptoms, suggesting a complex interplay between lipid metabolism and GI health in ASD.

Polyunsaturated fatty acids (PUFAs) have garnered increasing attention because of their significant roles in signal transduction (22). The present study also revealed abnormal PUFA metabolism. AA, an important ω-6 PUFA and a precursor of eicosanoids (23), is a major component of cell membranes and participates in neuronal signaling, synaptic plasticity, and neuroprotection, playing a crucial role in nervous system development and function (24). Although existing research on AA levels in children with ASD is not entirely consistent, most studies suggest decreased AA levels (6, 16) and dysregulation of AA metabolites, particularly prostaglandin E2 (13), indicating a significant role for AA in the pathogenesis of ASD. Although we did not observe differences in the ω-3/ω-6 ratio (25), we detected abnormalities in the ω-3 fatty acid/AA ratio. The relationship between ω-3 PUFAs and AA plays an antagonistic role in maintaining homeostasis (26). The intake of ω-3 PUFAs reduces AA concentrations, thereby downregulating the synthesis of AA-derived signaling mediators (eicosanoids) in cell membranes. DHA, EPA, and ALA (the precursors of EPA) are important members of the ω-3 fatty acid family. In this study, no differences were observed in the DHA/AA and EPA/AA ratios, but the ALA/AA ratio was significantly elevated compared with that in the control group, reflecting the enhanced competitive interaction between ω-3 fatty acids and AA in ASD patients, which may contribute to ASD development by further downregulating neuroprotective factors such as ceruloplasmin, superoxide dismutase, and transferrin (17). A randomized, double-blind, placebo-controlled trial revealed that daily supplementation with a high dose (250 mg) of AA significantly improved social interaction deficits in autistic children, possibly related to improved fatty acid ratios (27). However, this study did not collect a 1- or 3-day food log from the family to objectively assess the impact of dietary unsaturated fatty acids on plasma fatty acids, which needs further investigation.

According to the WHO guidelines for saturated fatty acid intake (28), the intake of saturated fatty acids in children should be less than 10% of the total energy. In fact, data from standard 3-day dietary records assessing nutrient intake in children have shown that children with ASD have lower saturated fatty acid intake than TD children do (29). While most studies report that the plasma levels of some saturated fatty acids in autistic children are higher than or not significantly different from those in TD children (15, 30, 31), this study revealed that the plasma C24:0 levels in autistic children were lower than in the control group, suggesting a complex mechanism of fatty acid metabolism in autistic children. C24:0 is an important component of myelinated nerve axons and synapses in the white matter of the brain, where it can constitute up to 48% of the fatty acids in glyco-and sphingolipids (32). These findings suggest a crucial mechanistic link between aberrant myelination and ASD pathology (33). However, further research is needed to clarify this phenomenon.

Children with autism often exhibit GI symptoms, which may significantly impact metabolism via the gut-brain axis (34, 35). This study also revealed that GI issues are notably more prevalent in children with autism than in TD children. Therefore, we further explored their interaction with GI symptoms. Although the interaction terms were not statistically significant, the Wald test indicated a potential association between GI status and altered fatty acid metabolism in children with autism. However, this association may be influenced by confounding factors such as dietary intake, which were not fully adjusted for in the current analysis. Further studies are needed to clarify the specific role of GI symptoms in these metabolic changes (36, 37).

Some limitations of this study warrant further discussion. First, the sample size included 70 ASD patients and 61 TD children, which may be insufficient to detect all potential differences in fatty acid metabolism. Future studies need to expand the sample size to confirm the fatty acid metabolic characteristics of children with ASD. Second, all three described fatty acids may be potential biomarkers for the diagnosis of autism, with AUC values significantly above 0.5 (38). However, the AUC values are only approximately 0.60, which means that they are not clinically adequate. This may be related to the insufficient sample size, and future studies with larger samples are needed for validation. The non-significant findings for other fatty acids should be considered exploratory and interpreted with caution. Third, this study was a single-center study. We observed that gastrointestinal symptoms in individuals with ASD affect fatty acid metabolism, which suggests that dietary habits in different regions may also have significant effects on fatty acid metabolism. Future multicenter studies with larger samples should clarify this. Fourth, gastrointestinal symptoms in this study were not diagnosed through clinical assessment, which may somewhat affect the interpretation of the results. Fifth, the lack of 1- or 3-day food log from the family prevented comprehensive assessment and adjustment of nutritional intake, despite implementing fasten protocols for blood collection and excluding participants receiving fatty acid supplementation at baseline. Lastly, while univariate methods were used for quality control, future studies would benefit from incorporating multivariate analyses (e.g., Principal Component Analysis) in the initial data exploration phase to provide a global overview and aid in outlier detection.

In conclusion, our findings provide a more detailed perspective on the role of fatty acids in ASD, particularly in the context of GI symptoms. These findings contribute to a deeper understanding of the metabolic underpinnings of ASD and suggest new avenues for research and therapeutic strategies.

## Data Availability

The original contributions presented in the study are included in the article/**Supplementary Material**. Further inquiries can be directed to the corresponding author.
