# Supplementary material for: Altered plasma fatty acids composition in autism spectrum disorder: a case-control study
Source: Front Psychiatry. 2025 Sep 24;16:1627704. doi: 10.3389/fpsyt.2025.1627704 (PMC12504180; doi:10.3389/fpsyt.2025.1627704)
Supplement: Supplementary file 1 [file Table1.docx]

**Supplementary File 1**

**Reagents**

37 fatty acid methyl esters mixture (see Table S1), 2,6-Di-tert-butyl-4-methylphenol (BHT), and acetyl chloride were analytical grade and purchased from Sigma-Aldrich (Sigma-Aldrich, Inc., USA). Heptadecanoic acid-D33 (C17:0-D33) was purchased from CDN (CDN Isotopes Inc., Canada). K2CO3 was analytical grade and purchased from SCRC (Sinopharm Chemical Reagent Co., Ltd., China). HPLC grade methanol was purchased from Merck (Merck KGaA, Darmstadt, Germany), and hexane was purchased from Thermo Fisher (Thermo Fisher Scientific Inc., USA).

**Metabolites extraction**

Sample preparation

Serum samples (10 μL) were mixed with 10 μL of the internal standard (0.2 mg/mL of C17:0-D33, 0.4 mg/mL of BHT in methanol) and 200 μL of methanol-hexane (4:1, v/v). The tubes were gently vortexed and then placed in a self-made liquid nitrogen bath for 10 min. 20 μL of acetyl chloride was added to the tubes slowly then kept in the dark at room temperature for 24 h. 0.5 ml of 6% K2CO3 solution was added slowly to stop the reaction and neutralize the mixture in an ice bath. After 50 μL of hexane was added to extract fatty acid methyl esters, and the tubes were briefly vortexed and then centrifuged (3000 rpm, 10 min), the top layer was collected and transferred to a sample vial for GC analysis.

GC-FID/MS Analysis

The GC-FID/MS consisted of an Agilent 7890B gas chromatography coupled to an Agilent 5977B mass spectrometer with a flame ionization detector (Agilent Technologies, USA). An Agilent DB-225 capillary GC column (10 m, 0.1 mm ID, 0.1 μm film thickness) was employed with sample injection volume of 2 μL and a splitter (1:20). Helium gas was used as carrier gas, nitrogen gas was used makeup gas. The injection port and detector temperatures were both set at 230 °C. The column temperature was set to 55 °C for 1 min and then increased to 205 °C with a rate of 30 °C/min. Colum temperature was then kept at 205 °C for 3 min and increased to 230 °C (5 °C/min), held at 230°C for 1 min. The MS spectra were acquired with the EI voltage of 70 eV and the m/z range of 45-450.

Data preprocessing

Peak determination and peak area integration were performed with MassHunter Workstation software (Agilent, Version B.08.00). Methylated fatty acids were identified by comparing with a chromatogram from a mixture of 37 known standards and further confirmed with their mass spectral data. The concentration of individual fatty acid was calculated from the FID data related to the internal standard.

Table S1: 37 fatty acid methyl esters and the deuterium-labeled internal standard

|  | **Abbreviation** | **Common Name** | **Fatty acid** |
| --- | --- | --- | --- |
| 1 | C4:0 | Butyric acid | Butyric acid |
| 2 | C6:0 | Caproic acid | Caproic acid |
| 3 | C8:0 | Caprylic acid | Caprylic acid |
| 4 | C10:0 | Capric acid | Capric acid |
| 5 | C11:0 | Undecanoic acid | Undecanoic acid |
| 6 | C12:0 | Lauric acid | Lauric acid |
| 7 | C13:0 | Tridecanoic acid | Tridecanoic acid |
| 8 | C14:0 | Myristic acid | Myristic acid |
| 9 | C14:1 | Myristoleic acid | *Cis*-9-tetradecenoic acid |
| 10 | C15:0 | Pentadecanoic acid | Pentadecanoic acid |
| 11 | C15:1 |  | *Cis*-10-Pentadecenoic acid |
| 12 | C16:0 | Palmitic acid | Hexadecanoic acid |
| 13 | C16:1 | Palmitoleic acid | *Cis*-9-Hexadecenoic acid |
| 14 | C17:0 | Heptadecanoic acid | Heptadecanoic acid |
| 15 | C17:1 |  | *Cis*-10-Heptadecenoic acid |
| 16 | C18:0 | Stearic acid | Octadecanoic acid |
| 17 | C18:1n9t | Elaidic acid | *Trans*-9-Octadecenoic acid |
| 18 | C18:1n9c | Oleic acid | *Cis*-9-Octadecenoic acid |
| 19 | C18:2n6t | Linolelaidic acid | *Trans*-9,12-Octadecenoic acid |
| 20 | C18:2n6c | Linoleic acid | *Cis*-9,12-Octadecenoic acid |
| 21 | C18:3n6c | γ-Linolenic acid | *Cis*-6,9,12-Octadecatrienoic acid |
| 22 | C18:3n3c | Linolenic acid | *Cis*-9,12,15-Octadecatrienoic acid |
| 23 | C20:0 | Arachidic acid | Icosanoic acid |
| 24 | C20:1 |  | *Cis*-11-Eicosenoic acid |
| 25 | C20:2n6 |  | *Cis*-11,14-Eicosadienoic acid |
| 26 | C20:3n6 | γ-Homolinolenic acid | *Cis*-8,11,14-Eicosatrienoic acid |
| 27 | C20:3n3 |  | *Cis*-11,14,17-Eicosatrienoic acid |
| 28 | C20:4n6 | Arachidonic acid (ARA) | *Cis*-5,8,11,14-Eicosapentaenoic acid |
| 29 | C20:5n3 | Eicosapentaenoic acid (EPA) | *Cis*-5,8,11,14,17-Eicosapentaenoic acid |
| 30 | C21:0 |  | Heneicosanoic acid |
| 31 | C22:0 | Behenic acid | Docosanoic acid |
| 32 | C22:1 | Erucic acid | *Cis*-13-Docosenoic acid |
| 33 | C22:2n6 |  | *Cis*-13,16-Docosadienoic acid |
| 34 | C22:6n3 | Docosahexaenoic acid (DHA) | *Cis*-4,7,10,13,16,19-Docosahexaenoic acid |
| 35 | C23:0 |  | Tricosanoic acid |
| 36 | C24:0 | Lignoceric acid | Tetracosanoic acid |
| 37 | C24:1 | Nervonic acid | *Cis*-15-tetracosenoic acid |
|  | C17:0-D33 | Heptadecanoic acid-D33 |  |
